# CABG versus CABG+TMR in patients with and without advanced CAD

**DOI:** 10.1186/1749-8090-10-S1-A89

**Published:** 2015-12-16

**Authors:** Ilya Berishvili, Marat Semenov, Peter Gusev

**Affiliations:** 1 TMR Department, Bakoulev Cardiovascular Scientific Center, Moscow, 199991, Russian Federation

## Background/Introduction

Intraoperative and hospital benefits from transmyocardial laser revascularization (TMR) may be related to acute sympathetic denervation. This study hypothesized that TMR as an adjunct to CABG would improve myocardial runoff in the TMR-treated regions and increase graft flow.

## Aims/Objectives

To compare the results of different operations (CABG versus CABG+TMR) in patients with and without advanced CAD and intraoperative comparison with changes on the microvascular level (spasm).

## Method

The results of 831 operations with CABG in patients with CAD in 3 groups were evaluated: CABG in patients without diffuse lesions of coronary arteries (group I - 711 patients), isolated CABG in patients with advanced CAD (group II - 33 patients) and CABG combined with TMLR in patients with advanced CAD (group III - 87 patients).

Data evaluation was made on the basis of the data of intraoperative EchoCG and coronaro-shuntography, the values of cardiac enzymes (CPK and CPK-MB) and data of intra- and postoperative evaluation, which allow valuing of patients condition.

## Results

Critical spasm of coronary arteries in group I occurred 1.7% of cases, in group II - 33.3%, in group III - 1.15%. Incidence of myocardial infarction was in group I 0,6%, in group II-20,3%, in group III -1,15%/.Mortality rate in group I was 2,8%, in group II -12,1%, in group III -1,15%/.

As patients in group I and II differed only in presence of diffuse changes of coronary arteries, it appears that development (and/or worsening) of critical spasm, high incidence of MI and mortality is prerogative of patients with advanced CAD. As patients in group II and III differ only in extent of operations, represented data show that the performance of TMLR in addition to CABG prevents development of critical spasm of coronary arteries and significantly improves effectiveness of operations.

## Discussion/Conclusion

Possibility of intraoperative spasm development of coronary arteries, is determined by diffuse changes of coronary arteries: TMLR, performed as an adjunct to CABG in patients with advanced CAD, ameliorates vasoconstriction (spasm), improves myocardial runoff (vasodilatation), acutely improves graft and coronary artery flow, and can significantly improve the results of operations.

